# The care needs of patients with idiopathic pulmonary fibrosis and their carers (CaNoPy): results of a qualitative study

**DOI:** 10.1186/s12890-015-0145-5

**Published:** 2015-12-04

**Authors:** Cathy Sampson, Ben Hope Gill, Nicholas Kim Harrison, Annmarie Nelson, Anthony Byrne

**Affiliations:** Cardiff University School of Medicine, Marie Curie Palliative Care Research Centre, Heath Park, Cardiff, CF14 4YS UK; Department Respiratory Medicine, University Hospital Llandough, Cardiff, UK; Swansea University, School of Medicine, Swansea, UK

**Keywords:** Idiopathic pulmonary fibrosis, Palliative care, Care pathways, Patient and carer experience, Key turning points

## Abstract

**Background:**

Idiopathic pulmonary fibrosis (IPF) is a chronic, fibrotic interstitial lung disease of unknown origin. It has a median survival of three years but a wide range in survival rate which is difficult to predict at the time of diagnosis. Specialist guidance promotes a patient centred approach emphasising regular assessment, information giving and supportive care coordinated by a multidisciplinary team (MDT). However understanding of patient and carer experience across the disease trajectory is limited and detailed guidance for MDTs on communication, assessment, and triggers for supportive and palliative interventions is lacking. This study addresses uncertainties relating to care needs of patients and carers at different stages of the IPF disease trajectory.

**Methods:**

Following ethical approval a multi-centre mixed-methods study recruited participants with IPF at four stages of the disease trajectory. Qualitative analysis was used to analyse 48 semi-structured interviews with patients (27) and paired carers (21).

**Results:**

Patients and carers outlined key elements of MDT activity capable of having significant impact on the care experience. These were structured around:

- Focus of clinical encounters
- Timely identification of changes in health status and functional activity
- Understanding of symptoms and medical interventions
- Coping strategies and carer roles.

**Conclusions:**

Patients diagnosed with IPF have a clear understanding of their prognosis but little understanding of how their disease will progress and how it will be managed. In depth analysis of the experiences of patients and carers offers guidance for refining IPF clinical pathways. This will support patients and carers at key transition points in line with National Institute for Health and Care Excellence (NICE) guidance.

**Electronic supplementary material:**

The online version of this article (doi:10.1186/s12890-015-0145-5) contains supplementary material, which is available to authorized users.

## Background

Idiopathic pulmonary fibrosis (IPF) is a progressive, life-limiting condition thought to arise from aberrant would healing following repeated alveolar microinjury, resulting in progressive lung fibrosis [1]. Epidemiological evidence suggests IPF incidence is rising by 5 % per annum in the UK, with over 4000 new cases and 3000 deaths each year [2, 3]. Whilst median survival for people with IPF is about three years, its clinical course is variable; in some patients lung function deteriorates slowly whilst others have a rapidly progressive course and occasional patients experience a particularly fulminant acute exacerbation resulting in precipitous decline [4]. Furthermore, at the time of diagnosis, it is difficult to accurately predict an individual’s disease trajectory leaving patients with an uncertain future.

**Fig. 1:**
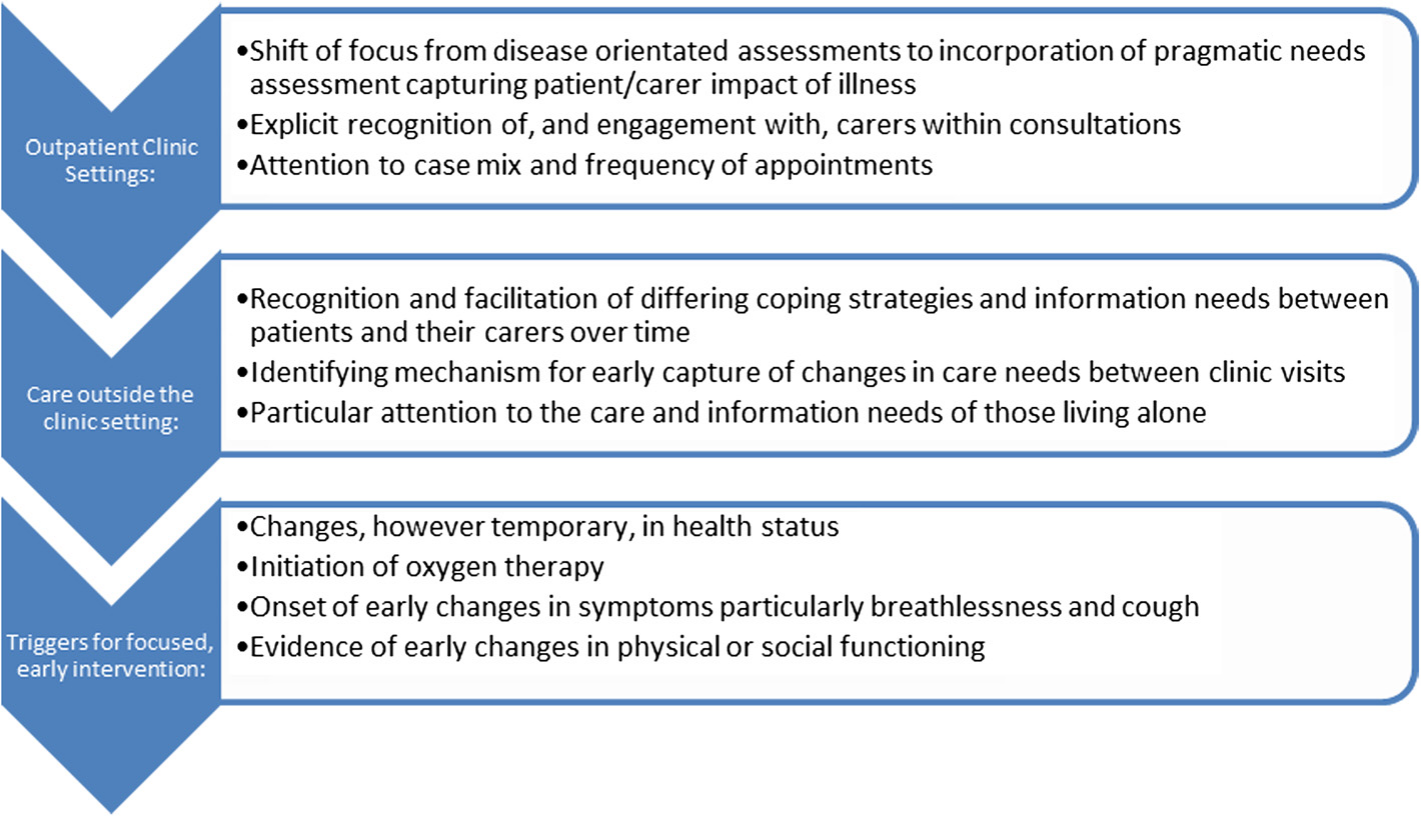
Implications for practice

There are few parameters that will accurately predict rate of decline at the time of diagnosis and the only therapy that improves survival is lung transplantation in eligible candidates.

Patients with IPF describe reduced health status, high symptom burden and impaired quality of life [5, 6]. They have a twofold higher use of inpatient and outpatient resources compared to the background population, and higher direct medical costs [7]. There are few interventions known to improve functional or symptomatic outcomes and a lack of consensus as to what outcome measures are important in clinical practice and research [8, 9]. Current guidance promotes a patient and carer centred, supportive approach coordinated by a multidisciplinary team with appropriate skill mix [10–12]. However, most research to date has focused on disease modification measured by respiratory function tests, with limited objective information on the wider impact of IPF on daily life. Furthermore, our understanding of patient and carer experience across the varied disease trajectories is limited, and there is no detailed guidance for MDTs on communication, assessing the need for supportive and palliative interventions, and identifying the triggers which should initiate them as the disease progresses.

Qualitative research provides rich data, giving insight into how people make sense of their experiences and helping elucidate complex settings and interactions. It plays an increasingly important role in health service research and is particularly helpful in understanding the supportive role of carers, whose perspective may not be appreciated in the clinical setting [13–16]. A small number of important qualitative studies of IPF have explored aspects of patient quality of life and experience of illness [17–19], but these have not compared patients at different stages of disease and there is little information on the experiences and needs of carers.

This study was designed to explore the perspectives of patients and their carers across the IPF spectrum to inform the development of clinical pathways and multidisciplinary service interventions as described in Table 1.

**Table 1:** Specific objectives of the CaNoPy study

1. Describe changes in individuals’ and carers’ perceived care needs at different stages of IPF in order to improve future service interventions
2. Identify time points or triggers at which supportive and palliative care services might effectively be introduced
3. Define what information individuals with IPF and their carers require over time
4. Evaluate the experiences and roles of carers for people with IPF

## Methods

### Study design

The uncertain nature of disease progression in IPF makes a longitudinal study difficult to achieve in a set time frame. Therefore, we chose a cross-sectional, mixed method design to study patients at different stages of IPF and matched carers. Specialists with expertise in palliative care and interstitial lung disease, who were part of the research team, generated a disease typology to classify patients into four stages of IPF described in Table 2. Patients were categorized according to disease extent: limited or extensive, and also disease behaviour: stable or progressive. This produced the four representative trajectories, for example limited stable or extensive progressive.

**Table 2:** IPF disease stage samples for CaNoPy

Disease extent	Limited	Extensive
	Limited disease: forced vital capacity (FVC) greater than 50 % predicted and gas transfer (TLCO) greater than 40 % predicted	Extensive disease: FVC less than 50 % or TLCO less than 40 % predicted
Disease behaviour	Stable	Progressive
	Stable disease: a decline of less than 10 % in FVC or less than 15 % in TLCO in the previous 12 months	Progressive disease: a decline in either FVC greater than 10 % or TLCO greater than 15 % during the previous 12 months

An experienced qualitative researcher (CS) conducted semi-structured interviews with participants and performed an initial analysis, using Interpretative Phenomenological Analysis (IPA). Emerging results were verified by other members of the research team. In keeping with recommendations for IPA, we aimed for a sample size of 6–10 patients and 6–10 carers per group, representing a perspective rather than a population. Consolidated criteria for reporting qualitative research (COREQ) guidelines were followed [20].

The study gained favourable opinion, 20/04/2012, from the South East Wales Research Ethics Committee, Panel B, 12/WA/0109 prior to the study commencing. The sponsor was Cardiff University (SPON 1088-12).

### Recruitment

Twenty seven patients with IPF and twenty one matched carers were recruited from two UK specialist interstitial lung disease clinics between October 2012 and August 2014. Patients were included if they met the American Thoracic Society criteria for IPF diagnosis [11]. No patients had been prescribed pirfenidone. Patients who did not have a carer, but who wished to participate, were included. A carer was defined as a person of the patient’s choice who contributed most to their care or, in the earlier stages of disease, provided emotional support. They were recruited after patients’ agreement and gave written informed consent. Exclusion criteria for participants were factors preventing communication, comprehension of study information or providing informed consent. Of the 27 patients 18 were male and 9 female with 6 male carers and 15 female carers. There were 3 male patients living alone and 2 female patients.

### Analysis

Anonymised transcripts were analysed by the qualitative researcher (CS) to capture patients and carer themes as described in Table 3. Analysis is described in detail in the online Additional file 1: Appendix. Table 4 outlines the analysis framework.

**Table 3:** Participant demographics

Patient category	Mean age	Age range	Gender	Lung transplant	Oxygen
Extensive Progressive	69.5	56–77	2 F 1 M	1	3
Limited Progressive	72.6	59–81	2 F 3 M	1	0
Extensive Stable	71	69–82	2 F 4 M	0	2
Limited Stable	75.1	66–87	3 F 6 M	0	0

**Table 4:** Interpretative phenomenological analysis framework

• Initial reading	
Reading of first transcript line-by-line, with preliminary comments	
• Early analysis	
Comments grouped into themes	
• Higher level abstraction	
Relationships developed between themes leading to an organised master list and thematic account of the case	
• Subsequent transcripts	
New themes tested against the previous transcripts as non-recurring themes were tested against following transcripts. Relationships across cases noted to identify a set of superordinate themes for the group	

## Results

Patients reported that their overall experience of living with IPF was one of coming to terms with a complex condition requiring a complicated and often protracted diagnostic work-up, with an uncertain but limited prognosis. They felt they received insufficient information on the clinical and practical management of their disease and would have welcomed more advice on pragmatic interventions at key points to help manage an unpredictable future. Although several common themes emerged for all patients, there was evidence of variation in the impact of IPF on patients from different disease trajectories.

Additional file 2: Box 1 summarises the key themes, common to patients and carers from all four trajectories, where IPF had significant impact on daily life.

### Communication and information

#### Communication skills

Patients and carers reported that their key needs were structured around context, timing, content and format of information. They described a need to balance honesty and hope, whilst dealing with a terminal prognosis but searched for positive ways to manage and live with an uncertain disease trajectory (Additional file 3: Box 2).

### Context

The specialist IPF clinic was seen as a trustworthy source of information. However, focus of the consultation was often perceived as disconnected from participants’ experience of the disease and its impact on everyday life. They felt unable to interpret the relevance of disease-focused assessments such as lung function tests to future exercise capacity or overall prognosis (Additional file 4: Box 3). Carers felt their role in clinical consultations was ambiguous and passive and this compounded the difficulty of accessing and interpreting information. Carer needs for independent information and advice were overshadowed by their desire to avoid distress to their partners in the clinic setting. Outside the specialist clinic the relative lack of knowledge about IPF in Primary Care added to the burden of uncertainty about future management.

#### Timing

The complexity of the diagnostic process often resulted in prolonged investigations and delays in referral to the appropriate specialist. Initial relief that the diagnosis was not cancer was replaced by shock at the likely prognosis. Patients and carers in the Limited Stable trajectory in particular struggled with uncertainties around the possible course of IPF. Their need to receive information at an appropriate pace triggered by changes they perceived in health status didn’t always coincide with the timing of clinic appointments. Paradoxically, they recalled feeling greatest concern about attending clinics where they had observed fellow patients with more severe IPF.

The specialist nurse was perceived as a key resource, enabling patients and carers to access help on practical management and triggering medical intervention when required. Despite this, patients and carers still struggled with uncertainty around key turning points if they occurred between clinic visits.

#### Content and format

Patients and carers appeared to show a good understanding of the overall prognosis for IPF but had difficulty translating this to their own particular disease progression and the management/support options available to them. They declared a need for specific information relating to oxygen therapy, nutrition, exercise, management of cough and breathlessness and disease management towards the end of life. Patients living alone were most direct in their need for information about future care planning.

Written information was most often suggested as useful. The internet was sometimes cited as portraying ‘worst case scenarios’ and there were mixed responses to the utility of a peer support group.

### Changes in health status

#### Monitoring disease progression

As patients and carers had difficulty interpreting objective, clinic-based disease assessments, they described how they developed their own subjective strategies for monitoring IPF. These included looking for clues to health status by observing alterations to the frequency of clinic appointments and comparison with other patients encountered in the clinic or support groups. They also associated deterioration of IPF with intercurrent illnesses such as chest infections, initiation or increased use of use of oxygen, and changes in functional activity (Additional file 5: Box 4).

#### Negotiating disease progression

Sustained deterioration in health status led subjects to re-evaluate the uncertainty of their future (Additional file 6: Box 5). For those in the progressive trajectories, these shifts were associated with emerging differences in the information needs and coping styles of their carers who sought frank, objective information about functional deterioration and prognosis to help plan future care. This contrasted with patient reluctance to receive such detailed information. Perhaps unsurprisingly, the clinic environment struggled to meet these differing needs.

### Functional activity

#### Diminished possibilities

Deterioration in health increased risk of social isolation for patients and carers, who experienced loss in relinquishing valued activities and responsibilities. Both groups adapted to a lifestyle of gradually declining choices rather than seeking assistance to optimise their functional activities (Additional file 7: Box 6). This was particularly apparent in patients living alone.

Most patients and carers felt unable to identify potential sources of help due to their uncertainty about the IPF course. Declining health status increased the emotional and domestic burden on carers, while patients expressed fears about employment loss, finance and how their partner would cope in future.

### Understanding of symptoms and medical interventions

#### Specific concerns

There were specific concerns, especially for carers, regarding symptom monitoring in relation to breathing, cough and use of oxygen. Differing expectations between patients and carers were particularly evident around oxygen use (Additional file 8: Box 7). Carers viewed it positively as facilitating daily living. However patients often interpreted continual use of oxygen as a failure on their part which undermined the carers’ attempts to improve functional activity inside and outside the home. Oxygen therapy highlighted the perceived disparity between objective assessment of health status in IPF and functional ability of patients in everyday life.

#### Medical management

Patients viewed pulmonary rehabilitation as positive intervention, enabling them to participate in the management of their own disease and improve everyday living through increased awareness of coping strategies like energy conservation and better task management. Carers felt these strategies were more readily accepted if offered by a professional rather than by themselves.

#### Palliative care

Patients and carers frequently compared their situation unfavourably with cancer patients whom they considered to have “help coming from every direction”. In this context, common misconceptions about palliative care emerged in relation to reasons for referral as did concerns about being referred onto “that path to death.”

Two patients had received palliative care, one in a hospice out-patient programme and the other through home visits. They reported significant improvement to quality of life through peer support, and expert advice on sleep problems, palliative medication and home adaptations.

### Roles and coping strategies of patients and carers

#### Patients

This study discovered significant differences in the range of coping strategies adopted by patients and carers. Core coping strategies for patients across all four trajectories included acceptance and adapting to change (Additional file 9: Box 8). The most significant factor for patients was whether they lived alone or had a partner. Patients living alone expressed their need for information about the future course of the disease and available sources of support.

Patients with partners expressed concern about how their partner would cope as the disease progressed.

#### Carers

Carers provided motivation and emotional support, placing the needs of the patient above their own (Additional file 10: Box 9). They were less likely to express positivity as a coping strategy for themselves and expressed feelings of not being prioritised and feeling unsure how to help. However, they did help implement strategies such as adaptation of lifestyle and task management to conserve energy, or using oxygen. The supportive role of the carer was particularly marked in the Extensive Progressive group, but carers across all trajectories expressed fears about managing their domestic situation in the future. Carers expressed living with feelings of guilt in all trajectories. The majority of carers in the study were female but male carers discussed how they had adapted gender roles to cope with the changing domestic situation as their partner deteriorated.

## Discussion

This is the first qualitative study to report the support needs of people suffering from IPF across defined disease trajectories, as well as providing an in-depth perspective on the needs of their carers. The results highlight specific opportunities to inform MDT assessments, case mix and resource use (Fig. 1).

A striking finding of the study is the identification of key communication strategies valued by patient and carers. Although difficulties accessing information have been previously described [18, 21], there has been little exploration of the discrete preferences of patients and carers to support practice change. By contrast, the present study identifies three areas for improvement. These are: to use the opportunity of clinical encounters to focus on supportive interventions and encourage aspects of self-management; to recognise carers as important participants in the consultation and finally, to appreciate that patients and carers differ in their needs for information and that these needs change over time.

The focus of clinic consultations on disease measures such as lung function appeared disconnected from participants’ lived experience of the disease and its impact on everyday life. They sought a more pragmatic needs assessment to include aspects of physical and social functioning, nutrition and symptom burden to support their self-management and guide their understanding of illness progression. This broader assessment approach has already been successfully employed in time pressured clinics in other settings including cancer [22].

The requirement to better acknowledge the role and needs of carers within outpatient consultations is an important finding. Carers described ambiguity in how they were perceived, with negative impact on both partners’ understanding of disease course and coping strategies. This implies a need for clinicians to change their perception of carers from passive observers to having active roles throughout the patient pathway. It also suggests that carers should have greater access to personal advice and support outside clinic settings, particularly at the time of diagnosis and during changes in health status.

An important difference between patients and carers is the finding that, although carers maintain positivity in their supporting role, they adopt much less positivity in coping strategies for themselves. The carers we interviewed felt they required a different balance in the personal support they receive from the IPF clinic and palliative care services, with particular attention to the social isolation and restriction of lifestyle that progressive IPF brings.

CaNoPy also identifies key milestones for study participants in their interpretation of disease progression. Changes in health status, even when temporary, produced significant shifts in coping. Changes in function, development of symptoms – particularly cough and breathlessness – and instigation of oxygen represented specific triggers for early and focused MDT support. Whilst Swigris [5] and Schoenheit [18] reported the impact of symptoms on health related quality of life, less attention has been paid to timing of assessments and triggers for intervention. In CaNoPy participants describe adapting to functional decline and symptom development, assuming ever decreasing choice in lifestyle rather than seeking assessment and support between clinic visits. This represents lost opportunity for early intervention in reducing symptoms and maintaining independence, as well as for efficient resource use.

The MDT is challenged to identify systematic approaches within the care pathway which facilitate earlier recognition of change. It underscores the need for a broader needs assessment and presents opportunities to examine strategies employed elsewhere, including the roles of care trackers and emerging technologies in supporting key workers [23–26].

Participants in CaNoPy who had received palliative care support identified improvements in quality of life by allowing them to achieve an adapted form of normal living. Instigation of oxygen therapy was a key turning point and an example of this type of adaptation. Whilst carers perceived this positively as an opportunity for maintenance of normal living, patients often felt it was stigmatic or an intervention to be used sparingly. Focused professional intervention at this point could maximise benefit and reinforce the legitimacy of carer strategies and patient involvement in self-management – which pulmonary rehabilitation achieved for those in receipt of it. Similarly carers appeared to take prime responsibility for management of symptoms, nutrition and exercise. It therefore seems likely that earlier access to palliative advice and interventions when symptoms first emerge may provide functional benefit and reduce carer anxiety and uncertainty.

It is well known that maintaining attitudes of positivity and hope are important when communicating with people suffering from IPF [19, 27, 28]. The present study extends this notion by identifying key differences in coping strategies adopted by patients at different stages of disease progression and between patients and carers. Patients in the Limited Stable group describe difficulty managing prognostic uncertainty, adopting a ‘coping day by day’ approach. Although they reported that meeting less well patients in IPF clinics or support groups was a threat to their ability to cope, they also felt isolated from support between clinic visits. This has implications for frequency of access (a suggestion that physiological stability does not equate to less need) and the casemix of clinics, where consideration is given to accommodating patients with similar disease behaviour. Patients living alone and those with extensive, progressive disease relied on accurate, honest assessment to help them plan for the future, challenging IPF clinics to recognise triggers for advance care planning.

### Limitations of this study

The clinic recruitment settings were within Wales, led by respiratory clinicians with a specialist interest in IPF: settings which may differ from other parts of the UK, including potential cultural differences and different service delivery models. Also, no patients interviewed were receiving pirfenidone, although findings should remain relevant to the majority of patients with IPF.

Although a longitudinal study design might be thought preferable, the cross sectional approach was used as the uncertain nature of disease progression made a longitudinal study difficult to achieve in the set time frame.

## Conclusions

Recent guidelines have defined the need for integrated clinical pathways in managing and supporting patients with IPF. This study provides new evidence of perceived gaps in the implementation of care across the disease trajectory and highlights the needs of carers in a detail not previously described. It suggests that in the outpatient context, a more practical needs assessment based on patient and carer perceptions is required, using tools which are both relevant and applicable to the clinic settings.

Greater consideration of clinic case mix and frequency of appointments is required, particularly for patients with stable disease. Clinical assessments should directly address implications for future care planning, mindful that patients and carers are likely to shift their expectations at different speeds.

Finally, this study highlights the importance of timing: identifying turning points more quickly in order to trigger timely assessment and intervention to improve patient and carer outcomes. Taken together our findings have important implications for the structure and function of IPF Clinics, multidisciplinary teams and associated clinical pathways.

## Additional files

Additional file 1:
**COREQ guidelines.** (DOCX 285 kb) Additional file 2:
**Box 1.** Key themes. (DOCX 11 kb) Additional file 3:
**Box 2.** Communication Skills. (DOCX 12 kb) Additional file 4:
**Box 3.** Context. (DOCX 13 kb) Additional file 5:
**Box 4.** Monitoring disease progression. (DOCX 12 kb) Additional file 6:
**Box 5.** Negotiating disease progression. (DOCX 13 kb) Additional file 7:
**Box 6.** Diminished possibilities. (DOCX 12 kb) Additional file 8:
**Box 7.** Specific concerns. (DOCX 12 kb) Additional file 9:
**Box 8.** Patients. (DOCX 11 kb) Additional file 10:
**Box 9.** Carers. (DOCX 13 kb)

## Abbreviations

COREQ: Consolidated criteria for reporting qualitative research
IPF: Idiopathic Pulmonary Fibrosis
IPA: Interpretative Phenomenological Analysis
MDT: Multidisciplinary team
NICE: National Institute for Health and Care Excellence
