# Supplementary material for: The care needs of patients with idiopathic pulmonary fibrosis and their carers (CaNoPy): results of a qualitative study
Source: BMC Pulm Med. 2015 Dec 4;15:155. doi: 10.1186/s12890-015-0145-5 (PMC4670492; doi:10.1186/s12890-015-0145-5)
Supplement: Additional file 1: — COREQ guidelines. (DOCX 285 kb) [file 12890_2015_145_MOESM1_ESM.docx]

**Appendix**

**Title:** The Care Needs of Patients with Idiopathic Pulmonary Fibrosis and their Carers (CaNopY): results of a qualitative study.

Sampson C, Hope Gill B, Harrison NK, Nelson A, Byrne A.

**Corresponding author:** Dr Anthony Byrne, Cardiff University School of Medicine, Marie Curie Palliative Care Research Centre, Heath Park, Cardiff CF14 4YS, United Kingdom.

[Anthony.Byrne2@wales.nhs.uk](mailto:Anthony.Byrne2@wales.nhs.uk)

Contact telephone number: 029 20687175

**Methods**

**Choice of method**

The role of qualitative research in capturing the perspectives of patients and carers makes it an essential component of the design and commissioning of healthcare services that best meets patient need [1]. The appropriate analysis of collected data is an essential part of this process [2]. Interpretative Phenomenological Analysis (IPA) was chosen as the best method to capture the experiences of patients and carers living with the incompletely understood terminal condition of Idiopathic Pulmonary Fibrosis (IPF). It is also interpretative in the sense that the researcher’s conceptions, and experience, as brought to the analysis, are recognised. The data for this study was collected from digitally voice recorded semi-structured interviews and all interviews were carried out by the same researcher (CS). Field notes were taken after each interview. The interview technique was an iterative process with each interview building on the recognition of themes of interest from the previous. IPA aims to explore the processes by which participants make sense of their experiences [3].

**Sample size**

Initial considerations related to choice of IPA and the sample size. Although the total sample size in the CaNoPy study is large, there are eight sub-groups, allowing in-depth analysis of all viewpoints.

| **Table 1: IPF disease stages**  1. Limited disease: forced vital capacity (FVC) greater than 50% predicted and gas transfer (TLCO) greater than 40% predicted;  2. Extensive disease: FVC less than 50% or TLCO less than 40% predicted;  3. Progressive disease: a fall in either FVC greater than 10% or TLCO greater than 15% during the previous 12 months;  4. Stable disease: a fall of less than 10% in FVC or less than 15% in TLCO in the previous 12 months. |
| --- |

Within this 6-8 paired patient carer groups were recruited, with the aim of exploring the experiences of the carer in depth in addition to the patient experience of IPF.

In practice there were three variations on the patient-carer dyad: where the patient and carer gave separate interviews; where the patient lived alone; and where patients and carers asked to be interviewed together. Each of these offered a valid perspective but required methodological consideration regarding adequate representation of the different perspectives during analysis.

IPA does not preclude the use of large numbers but, as with all qualitative research, does not claim generalisations [4]. The approach taken in the Canopy study follows the recommendation that patterns and relationships across groups should be explored following the idiographic stage [5].

**Validity**

Group results were analysed by the qualitative researcher for consistent themes. A coding framework for emergent themes was developed by the qualitative researcher and then validated and compared by the research team. Any significantly different themes occurring between treatment groups were allocated comparatively.

**Analysis**

IPA is based on an idiographic approach beginning with a single case as a basis to develop more general categories developed in a detailed case-by-case analysis. The transcripts were systematically analysed in several stages by the qualitative researcher. Field notes were made as soon as possible after each interview in order to capture non-verbal communication and general impressions. These were used to inform the context of analysis but were not used formally.

Four separate projects were set up in NVivo to reflect the 4 stages of the disease trajectory. The final stage of cross comparison across stages was carried out manually. Analysis resulted in key emergent themes, called turning points, across the IPF trajectory. These encompassed all four stages, with emergent processes giving insight into how patients and carers negotiated these turning points, and additional information outlining any key differences between groups. A range of themes is presented to indicate the full spectrum of issues raised by patients and carers. A core range of issues is then shown to indicate those issues that are common across all four stages, in accordance with IPA principles of recurrence.^5^

**References**

1. Barker K. **How Can Qualitative Research Be Utilised in the Nhs When Re-Designing and Commissioning Services?** *British Journal of Pain* 2014;9(1):71-72.Barker 12014

2. Coulter A, Locock L, Ziebland S, Calabrese J. **Collecting Data on Patient Experience Is Not Enough: They Must Be Used to Improve Care.** *BMJ* 2014;348:g2225 doi: 10.1136/bmj.g2225.

3. Brocki J and Wearden A. **A Critical Evaluation of the Use of Interpretative Phenomenological Analysis (IPA) in Health Psychology.** *Psychology and Health* 2006;**21**(1):87-108.

4. Smith J. (ed) *Qualitative Psychology. A Practical Guide to Research Methods.* London: Sage.

5. Smith J, Flowers P, Larkin M. *Interpretative Phenomenological Analysis: Theory, Method and Research*. London: Sage.

**Results overview**

**Key Themes and Processes**

The diagnosis of IPF challenged assumptions about the future. Patients and carers understood the terminal prognosis but had little idea of how they would navigate the IPF journey, characterised by a general lack of knowledge and information.

There were five main themes across the four stages of IPF, structured as key turning points for patients and carers where the IPF trajectory intersected with everyday life and function. These turning points of Communication and Information; IPF course; IPF and Everyday life; IPF Management and IPF Roles and Coping strategies and were united by the overarching theme of uncertainty and are presented within the three main processes by which patients and carers sought to re-orientate, minimize uncertainty and maximize their abilities to live with an incurable condition.

Patients and carers reclassified communication and information around developing a better understanding of IPF, reframed their new situation through adapting roles, coping strategies and activities of daily life, and redefined what the future trajectory of IPF might be through evaluating symptoms and interventions. Key differences between the four IPF disease stages were allocated as sub-themes within the main themes and developed insight into specific needs within the stages.

**
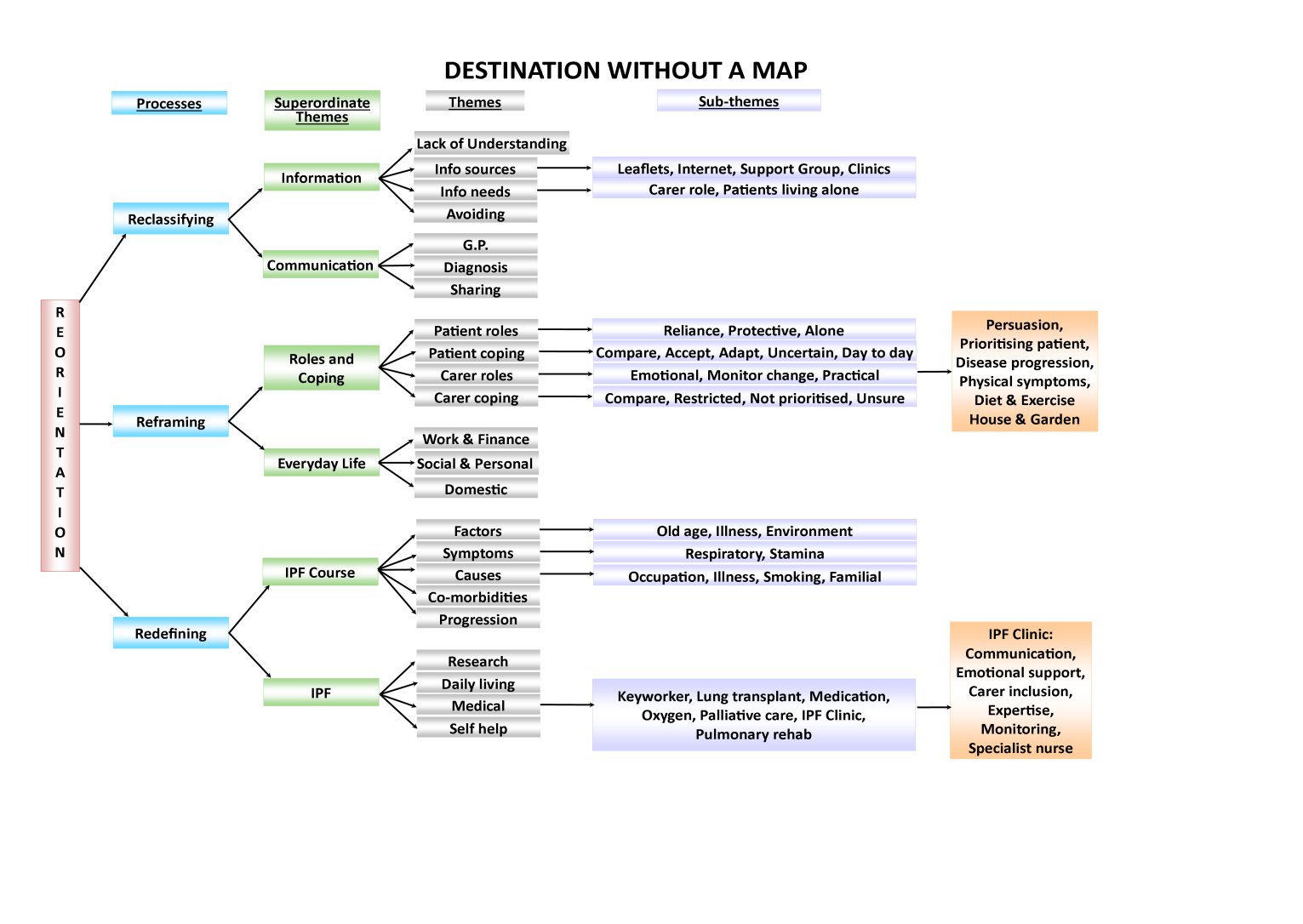
**

**Data extracts**

**Communication and Information**

**Illustrative data extracts following main paper themes**

**Communication skills**

**Carer: Extensive Stable**

Now, you don’t want somebody to say you’ve got six months to two years to live, but there’s an outside chance you might last ten…when I questioned [IPF consultant] further about it, I said well what is the longest a patient in this condition has lasted, one of your patients, [IPF consultant] then said ten years. Which I thought, oh why give her [patient] six months to two year when you actually have people who have, with the same set of circumstances have gone ten years. And that puzzled me a bit…

**Carer: Extensive Progressive**

… knowing that there’s nothing that can be done, it’s a progressive illness. We don’t… We, we’ve not been abandoned [by IPF clinic]. That’s what I mean by attention, we’ve not been abandoned.

**Patient: Extensive Progressive**

I’ve been told enough in the [IPF] clinic so you know… I’ve got copies from all the letters from the clinic as well, you know. I insisted I have a copy of them … I want to see what they are telling the GP, you know to make sure and to see what… to try and understand the diagnosis as well that is there, you know. That’s the main thing.

**Communication and Information**

**Context**

**Patient: Limited Progressive**

I spoke to the nurse and the consultant, the first time I went to the hospital and asked really straight questions, like ‘What is the prognosis?’

‘Between 3 and 5 years’.

‘What are the symptoms going to be?’

‘Well all of a sudden you’ll get worse and then you might get better’.

Nobody could give me any idea what it was going to be…and I said ‘What can you do for me?’

‘Nothing, we’re not going to give you anything for it.’

…Everytime I go back… I’ll ask the same questions and I’ll get the same answers…

**Carer: Limited Stable**

…it’s hard to anticipate what help I need if I don’t know what’s coming… I never know how doctors feel about a spouse sitting there and suddenly taking over the conversation so I generally don’t ask questions. I answer questions if I’m asked…but I think that I’m there just as a support system to [partner], rather than having my own questions answered.

**Timing**

**Patient: Limited Progressive**

… the feeling that there was the time and the space to explore the issue. I think that was the main thing. It was a question of not only having the process laid out for you which would have helped if that had been clearer, but also at what points in that process… if he [IPF consultant] couldn’t have dealt with it then, when I could have sat down with him and had that conversation… The second thing would have been actually information in your hand that you can take away. What does it mean? Once someone says you have eighteen months, what do you do? Now for me I have a lot of support, lot of strength from my partner, family. For others it won’t be there… so some indication of where in a very short period of time you could go to for some sort of help…

**Carer: Limited Stable**

… It’s all iffy isn’t it? … there’s nothing sort of concrete about it. It’s all sort of pie in the sky at the moment…life just goes on.

**Content and Format**

**Carer: Extensive Stable**

Handouts maybe… An explanation that you can take away and once the initial shock has worn off you can actually look at it without being dependant on the internet because there are so many different websites. There’s so much information that I think it’s an overload. Whereas if you’ve given a manageable amount of information you can digest that and then go away and look if you want to know more.

**Patient: Limited Progressive**

Well I knew somebody that’s got this disease… and from what I can understand he’s reasonable sitting in a chair, but other than that it’s hopeless…he’s got oxygen permanently and he’s got a stair-lift to go up to bed. Now he’s got a wife so he obviously has someone to take care of him but I’m widowed so don’t have anyone here, so you know when you get to that stage that’s the thing that I find a little concerning…

**IPF Course: Changes in health status**

**Monitoring disease progression**

**Carer: Limited Stable**

…when you’re told we don’t want to see you for a year that’s a wonderful sign… you come out of there dancing.

**Patient: Extensive Progressive**

It’s got quite a bit worse. It’s a weird thing ‘cos first of all it’s slow and then what it does it jumps down and it suddenly goes really bad. Usually with a bad chest infection. And you come out of it but you never quite recover back to where you were… And they’ve obviously been observed in the hospital like. Every time I have a lung function test they’ve seen it going down and down and down as it’s progressing.

**Carer: Extensive Stable**

I think at the end of any test they perform…let’s compare it with someone who is the norm… if it remains the same next time out and we’ve every hope that it will, well how nice to see that you’re holding the norm, that you are getting closer to the norm. And that fills us full of hope, and with hope there is every chance But we’re not stupid enough to be in denial to say this thing goes to go away .We know we’ve got to deal with it and the end result may be that I lose [patient].

**Negotiating disease progression**

**Carer: Extensive Progressive**

[Patient] can’t walk, so I didn’t know if maybe the oxygen would help him. I would like to know that. And I think [patient] thinks, once he’s gone on that… I think he thinks that’s the beginning of the end. That it’s a new stage. That it’s not the beginning of being able to do more. I would look at it as a beginning to be able to do more and I think he looks at it as the beginning of the end. That you’ve given in to something maybe or… Because it’s not… if it’s going to help you, it’s not giving in, is it?

And I daren’t talk about it too much to him because you don’t want to worry him. So it’s quite difficult.

**Patient and Carer: Extensive Progressive**

Carer: We’ve just pretended it wasn’t there almost in a way haven’t we? We’ve carried on as normal.

Patient: Well it almost wasn’t there to be honest.

Carer: It’s come as more of a shock I suppose hasn’t it, that you’ve gone down rather suddenly?

Patient: No. I accepted it because I knew here we go, this is all part and parcel of it, so it wasn’t a shock, it’s a disappointment that I can’t do things in a way that I would…And so it’s adapting, that type of thing.

Carer: I’ve certainly in the last six months…my vision of the future has changed quite markedly and…I’ve had to come to terms with the fact that it’s not going to be as I thought it was possibly going to be. And I think that’s been quite sudden. So I wouldn’t say I was shocked because I knew what was going to happen but I think I thought it would be a little bit more gradual where it seems to have been very much that you’ve gone like that.

Patient: Well the only thing I would say in relation to that is maybe it’s like a staircase that one’s on the flat bit at the top of the landing, one goes down, is it a continuing drop or would you stay there for a little while and another dip in another eighteen months or so?

**Patient and Carer: Limited Progressive**

Carer: …but we got to look at the part where it’s not always going to be like that is it? …As he gets older obviously things are going to change and they’re not going to change for the better are they? They are going to get worse.

Patient: There may be a tablet by then

Carer: Maybe so. It’s called the wonder drug.

**Carer: Extensive Progressive**

I’d like to know more about it. And maybe without [patient] being there, I would like to know what can happen at the end… I don’t want to sit with [patient] and know that. To see that I am devastated.

**Functional activity**

| **Diminished possibilities**  **Carer: Extensive Progressive**  And he’s not as fit so I find I’m not doing as much so I’m putting weight on…’Cos we used to go swimming together and now we’re not going swimming. And we used to go walking for the whole day and now we’re not… So I have out weight on and I know it’s not his fault but half of it is. It’s not his fault, it’s my fault because I’m eating more but maybe now in the afternoon I’m going upstairs and watching the television and having a cappuccino whereas before we would go out. We’d go out all the time.  **Patient: Extensive Stable**  But I have been quite lucky. I do manage to cope because what I can’t do one day I got to wait for a day when I can do it… shopping and things like that and if it’s really cold I got to be really careful… I’m lucky I got the car and I’m still able to drive to local shops. But I don’t go long distances now  **Carer: Limited Stable**  Physically it slows [patient] down a lot. We don’t go out as much as we used to. But we try to go for a walk to keep him agile but we’ve got to stop when he’s gone half a mile. We got to stop so he can get his breath. He can’t breathe. And we do it like that, see how far he can go without having to stop. |
| --- |

**Understanding of symptoms and medical interventions**

**Specific concerns**

**Carer: Limited Stable**

… the one thing I would want to say is the effect of the coughing, the constant coughing on others. It’s wearing… It’s the fear, it’s the worry [patient] coughs so much at night that a couple of years ago I moved into another bedroom. Cos I wasn’t getting any sleep…but it’s almost like being um a parent you know…You’re almost always on alert.

**Carer: Extensive Stable**

I say we need to ring the doctor then and we’ll make an appointment and he [patient] says well it’s not as bad now. I said well tough, I said. We’ve made that appointment I said, we’re not cancelling. We’ll just go and get it checked out and that. So it’s things like that sort of thing that you know sometimes you’re never quite sure and I think as his lung condition progresses where he could become more semi-invalid or whatever sort of thing, does the health service then sort of give you sort of options about the places you can have maybe a bit of support and help with different things?

**Carer: Extensive Stable**

He’s [patient] a bit pig headed with regards to the fact that he’s been given an oxygen tank now and he doesn’t like wearing that… and he seems to think he doesn’t need it at times but I keep on trying to say you do and that because this is what helps and it doesn’t put the pressure on to your heart once you’re sort of breathing correctly and getting that oxygen in which is what you need…

**Patient: Extensive Stable**

I have an oxygen pack … when I first started using it out or at functions I was very aware that people were staring at me. … Well what does it matter if they stare at me? I’m getting over that but it was difficult at first.

**Carer: Extensive Progressive**

… because he [patient] looks alright when he’s sitting, because he hardly… he doesn’t use very much of the oxygen at all. And I don’t know whether I should be saying, put it on and push yourself and go or whether I shouldn’t... and not be saying that, whether I should just let him… which is what I do. I let him decide what he wants to do and that’s what he does. And he definitely seems to want to use it less rather than more. Because I think he feels it’s a failure or that he’s, perhaps that he’s getting worse if he uses it. But I said to him, well you know, that’s what they said, when you move around you need the oxygen.

**IPF and Everyday Life**

**Key themes**

**IPF medical management**

**Medical management**

**Patient: Extensive Progressive**

… I think going on this pulmonary rehabilitation course. That was incredible. Fantastic that was, because not only did we have the dietician there, which OK, fine my diet anyway is not as bad as most people’s. And the physiotherapist there, very good guy because he actually talked about things and we got to know what we needed to do and I was the only one with IPF on the course. The others had COPD and various other things. They gave us a booklet we could read you know for the, for the course. And that was good and it gave us exercises to do.

**Patient: Extensive Progressive**

I’ve been put forward for lung rehabilitation… Just to keep me at this level of fitness… to be eligible for this lung transplant obviously… It’s basically exercise. And obviously a lot of things to do with diet and that sort of stuff.

**Carer: Extensive Stable**

It’s just you know, he [patient] likes to do things himself. He’s beginning to do things a bit more… He’s thinking he can’t do things sometimes. I think this six week course he’s had has really helped psychologically… But I think he’s realising now he can be doing things and that even though he can do them at a shorter pace, sit down and get a chair and things like that. I think he’s getting better at it now…

**Carer: Extensive Progressive**

I think it might have been nice if they had invited the partners along to listen because it was a course [pulmonary rehab] for him for exercise but then he had talks as well. Well it might have been nice for you to go and listen. I would be quite interested because you are in the dark about it.

**Palliative care**

**Patient: Limited Stable**

He [palliative care worker] comes along every couple of weeks or whatever. He’s been very good and very, very informative with the information and help that’s available and things.

**Patient: Limited Stable**

What worries me is my age, because they going to look at you, they don’t care how fit you are, they look… he’s seventy-seven, send him on that path to death. You know that path they got.

**Patient: Extensive Progressive**

It surprised me the first day. I thought they’ve all got cancer but I haven’t. So what am I doing here? [day hospice group].

**Patient and carer roles and coping strategies**

**Carers**

**Carer: Limited Stable**

…it’s hard to anticipate what help I need if I don’t know what’s coming… I never know how doctors feel about a spouse sitting there and suddenly taking over the conversation so I generally don’t ask questions. I answer questions if I’m asked…but generally speaking I think that I’m there just as a support system to [patient], rather than having my own questions answered.

**Carer: Extensive Progressive**

I go out with [relative] on a Saturday morning. I mean I feel guilty for going but [patient] says for goodness sake, go out for an hour. You need to go out for an hour. So I do.

… You do feel guilty, don’t you?... I’ve got to, to keep me sane. Because there isn’t, there isn’t anything to look forward to you know? I don’t look forward to any more than the day.

And that’s it. And then there’s the next day. And then there’s the next day and I don’t think

of anything else other than that day…I mean I do, I must admit, I do try and think what it’s going to be like and then I push it away. No, don’t want to know.

**Carer: Limited Progressive**

It’s the feeling of you know, what, is is there, you know I wish there was something you could tell me what I could do to assist rather than just sitting there you know and actually asking, can I help makes it worse I think because he just becomes even more anxious then. So I tend to sit there and say nothing and I’m thinking this is awful, I’m sitting here doing nothing and saying nothing because I don’t know quite what to do or say that would be of any assistance you know.

**Carer: Extensive Stable**

They told me nobody knows how it will progress … [patient] could be fine for quite a number of years or he could deteriorate rapidly… but where can you turn to if you needed somebody… maybe to talk to as you say, like a support group or a carer’s sort of support group or something like that. Or even you know they have these forums and different things.

**Patients**

**Limited Progressive**

I just sort of realised that it was a progressive disease and had degrees of severity, you know in different individuals … you’ve just got to accept it really haven’t you know. You’re getting older and not a lot is known about how it originates from what I understand so.

**Extensive Stable**

I do my jobs according to how I feel… Like cleaning one window one day and the next window the next day only if I feel like doing it. It’s working for me at the moment. If I should get any worse and I don’t know what would happen, that’s how I live. I adapt.

**Limited Progressive**

I don’t see that there would be any help in, if there’s no cure for it [IPF], in talking about it. I don’t see that that can help, like people trying to get inside your mind. I’ve never been one for that and I’m very private

**Extensive Stable**

If my wife can’t cope, who do I get in to help support me? Who have you referred me on to who will know of my case so that when I get to a point where I might need a hospice, is someone doing to approach me or am I going to have to chase around for that? All of those bits are not kind of considered. Human nature being what it is, I can understand why people don’t like talking about death. People don’t like talking about dying …because I can imagine there are some people who wouldn’t want them to tell them anything and others like myself who would much rather the opportunity of planning … so there are lots of areas of support that could be covered that aren’t… they are around prioritisation within the hospital.
