# Supplementary material for: The care needs of patients with idiopathic pulmonary fibrosis and their carers (CaNoPy): results of a qualitative study
Source: BMC Pulm Med. 2015 Dec 4;15:155. doi: 10.1186/s12890-015-0145-5 (PMC4670492; doi:10.1186/s12890-015-0145-5)
Supplement: Additional file 2: — Box 1. Key themes. (DOCX 11 kb) [file 12890_2015_145_MOESM2_ESM.docx]

PULM-D-15-00026R1

The Care Needs of patients with Idiopathic Pulmonary Fibrosis and their Carers (CaNoPy): results of a qualitative study.

**Box 1: Key themes**

| - Communication and information - Changes in health status - Functional activity - Understanding symptoms and medical interventions - Patient and carer roles and coping strategies |
| --- |
