# Supplementary material for: The care needs of patients with idiopathic pulmonary fibrosis and their carers (CaNoPy): results of a qualitative study
Source: BMC Pulm Med. 2015 Dec 4;15:155. doi: 10.1186/s12890-015-0145-5 (PMC4670492; doi:10.1186/s12890-015-0145-5)
Supplement: Additional file 3: — Box 2. Communication Skills. (DOCX 12 kb) [file 12890_2015_145_MOESM3_ESM.docx]

PULM-D-15-00026R1

The Care Needs of patients with Idiopathic Pulmonary Fibrosis and their Carers (CaNoPy): results of a qualitative study.

**Box 2: Communication skills**

**Carer: Extensive Progressive**

… knowing that there’s nothing that can be done, it’s a progressive illness. We don’t… We, we’ve not been abandoned [by IPF clinic]. That’s what I mean by attention, we’ve not been abandoned.

**Patient: Extensive Progressive**

I’ve been told enough in the [IPF] clinic so you know… I’ve got copies from all the letters from the clinic as well, you know. I insisted I have a copy of them … I want to see what they are telling the GP, you know to make sure and to see what… to try and understand the diagnosis.
