# Supplementary material for: The care needs of patients with idiopathic pulmonary fibrosis and their carers (CaNoPy): results of a qualitative study
Source: BMC Pulm Med. 2015 Dec 4;15:155. doi: 10.1186/s12890-015-0145-5 (PMC4670492; doi:10.1186/s12890-015-0145-5)
Supplement: Additional file 4: — Box 3. Context. (DOCX 13 kb) [file 12890_2015_145_MOESM4_ESM.docx]

PULM-D-15-00026R1

The Care Needs of patients with Idiopathic Pulmonary Fibrosis and their Carers (CaNoPy): results of a qualitative study.

**Box 3. Context**

**Patient and Carer: Extensive Stable**

Patient: I remember I used to come from there thinking, well how can they say it’s normal when I know it’s not… that’s the sort of feeling I got … but when I used to use the machine one thing and another everything seemed to be alright… I thought to myself, well how can that be when I know what I am feeling like.

Carer: I mean the thing is I think if they catch him on a good day… you know you get good days and bad days…

**Carer: Limited Stable**

…it’s hard to anticipate what help I need if I don’t know what’s coming… I never know how doctors feel about a spouse sitting there and suddenly taking over the conversation so I generally don’t ask questions. I answer questions if I’m asked…but I think that I’m there just as a support system to [partner], rather than having my own questions answered.
