# Supplementary material for: The care needs of patients with idiopathic pulmonary fibrosis and their carers (CaNoPy): results of a qualitative study
Source: BMC Pulm Med. 2015 Dec 4;15:155. doi: 10.1186/s12890-015-0145-5 (PMC4670492; doi:10.1186/s12890-015-0145-5)
Supplement: Additional file 5: — Box 4. Monitoring disease progression. (DOCX 12 kb) [file 12890_2015_145_MOESM5_ESM.docx]

PULM-D-15-00026R1

The Care Needs of patients with Idiopathic Pulmonary Fibrosis and their Carers (CaNoPy): results of a qualitative study.

**Box 4. Monitoring disease progression**

**Carer: Limited Stable**

…when you’re told we don’t want to see you for a year that’s a wonderful sign… you come out of there dancing.

**Carer: Extensive Stable**

I think at the end of any test they perform…let’s compare it with someone who is the norm… if it remains the same next time out and we’ve every hope that it will, well how nice to see that you’re holding the norm…And that fills us full of hope, and with hope there is every chance. But we’re not stupid enough to be in denial to say this thing goes to go away .We know we’ve got to deal with it and the end result may be that I lose [patient].
