# Supplementary material for: The care needs of patients with idiopathic pulmonary fibrosis and their carers (CaNoPy): results of a qualitative study
Source: BMC Pulm Med. 2015 Dec 4;15:155. doi: 10.1186/s12890-015-0145-5 (PMC4670492; doi:10.1186/s12890-015-0145-5)
Supplement: Additional file 6: — Box 5. Negotiating disease progression. (DOCX 13 kb) [file 12890_2015_145_MOESM6_ESM.docx]

PULM-D-15-00026R1

The Care Needs of patients with Idiopathic Pulmonary Fibrosis and their Carers (CaNoPy): results of a qualitative study.

**Box 5: Negotiating disease progression**

**Carer: Extensive Progressive**

[Patient] can’t walk, so I didn’t know if maybe the oxygen would help him. I would like to know that. And I think [patient] thinks, once he’s gone on that… I think he thinks that’s the beginning of the end. That it’s a new stage. That it’s not the beginning of being able to do more. I would look at it as a beginning to be able to do more and I think he looks at it as the beginning of the end. That you’ve given in to something maybe or… Because it’s not… if it’s going to help you, it’s not giving in, is it?

And I daren’t talk about it too much to him because you don’t want to worry him. So it’s quite difficult.

**Patient and Carer: Extensive Progressive**

Carer: We’ve just pretended it wasn’t there almost in a way haven’t we? We’ve carried on as normal.

Patient: Well it almost wasn’t there to be honest.

Carer: It’s come as more of a shock I suppose hasn’t it, that you’ve gone down rather suddenly?

Patient: No. I accepted it …this is all part and parcel of it, so it wasn’t a shock, it’s a disappointment that I can’t do things in a way that I would…it’s adapting, that type of thing.

Carer: I’ve certainly in the last six months…my vision of the future has changed quite markedly and…I’ve had to come to terms with the fact that it’s not going to be as I thought it was possibly going to be. And I think that’s been quite sudden. So I wouldn’t say I was shocked because I knew what was going to happen but I think I thought it would be a little bit more gradual where it seems to have been very much that you’ve gone like that.

Patient: Well the only thing I would say in relation to that is maybe it’s like a staircase that one’s on the flat bit at the top of the landing, one goes down, is it a continuing drop or would you stay there for a little while and another dip in another eighteen months or so?

**Carer: Extensive Progressive**

I’d like to know more about it. And maybe without [patient] being there, I would like to know what can happen at the end… I don’t want to sit with [patient] and know that. To see that I am devastated.
