# Supplementary material for: The care needs of patients with idiopathic pulmonary fibrosis and their carers (CaNoPy): results of a qualitative study
Source: BMC Pulm Med. 2015 Dec 4;15:155. doi: 10.1186/s12890-015-0145-5 (PMC4670492; doi:10.1186/s12890-015-0145-5)
Supplement: Additional file 7: — Box 6. Diminished possibilities. (DOCX 12 kb) [file 12890_2015_145_MOESM7_ESM.docx]

PULM-D-15-00026R1

The Care Needs of patients with Idiopathic Pulmonary Fibrosis and their Carers (CaNoPy): results of a qualitative study.

**Box 6. Diminished possibilities**

**Carer: Extensive Progressive**

And he’s not as fit so I find I’m not doing as much so I’m putting weight on… ‘Cos we used to go swimming together and now we’re not going swimming together. And we used to go walking for the whole day and now we’re not… So I have put weight on and I know it’s not his fault but half of it is. It’s not his fault, it’s my fault because I’m eating more but maybe now in the afternoon I’m going upstairs and watching the television and having a cappuccino whereas before we would go out. We’d go out all the time.

**Carer: Limited Stable**

Physically, it slows [patient] down a lot. We don’t go out so much as we used to. But we try to go for a walk to keep him agile…but he’s got to stop when we gone half, no, half a mile we got to stop and get his breath. He can’t breathe. And we do it like that, see how far he can go without having to stop.
