# Supplementary material for: The care needs of patients with idiopathic pulmonary fibrosis and their carers (CaNoPy): results of a qualitative study
Source: BMC Pulm Med. 2015 Dec 4;15:155. doi: 10.1186/s12890-015-0145-5 (PMC4670492; doi:10.1186/s12890-015-0145-5)
Supplement: Additional file 8: — Box 7. Specific concerns. (DOCX 12 kb) [file 12890_2015_145_MOESM8_ESM.docx]

PULM-D-15-00026R1

The Care Needs of patients with Idiopathic Pulmonary Fibrosis and their Carers (CaNoPy): results of a qualitative study.

**Box 7. Specific concerns**

**Carer: Extensive Progressive**

… because he [patient] looks alright when he’s sitting, because he hardly… he doesn’t use very much of the oxygen at all. And I don’t know whether I should be saying, put it on and push yourself and go or whether I shouldn’t... and not be saying that, whether I should just let him… which is what I do. I let him decide what he wants to do and that’s what he does. And he definitely seems to want to use it less rather than more. Because I think he feels it’s a failure or that he’s, perhaps that he’s getting worse if he uses it. But I said to him, well you know, that’s what they said, when you move around you need the oxygen.
