# Supplementary material for: The care needs of patients with idiopathic pulmonary fibrosis and their carers (CaNoPy): results of a qualitative study
Source: BMC Pulm Med. 2015 Dec 4;15:155. doi: 10.1186/s12890-015-0145-5 (PMC4670492; doi:10.1186/s12890-015-0145-5)
Supplement: Additional file 9: — Box 8. Patients. (DOCX 11 kb) [file 12890_2015_145_MOESM9_ESM.docx]

PULM-D-15-00026R1

The Care Needs of patients with Idiopathic Pulmonary Fibrosis and their Carers (CaNoPy): results of a qualitative study.

**Box 8. Patients**

| **Patient: Limited Progressive living alone**  Well I knew somebody that’s got this disease… and from what I can understand he’s reasonable sitting in a chair, but other than that it’s hopeless…he’s got oxygen permanently and he’s got a stair-lift to go up to bed. Now he’s got a wife so he obviously has someone to take care of him but I’m widowed so don’t have anyone here, so you know when you get to that stage that’s the thing that I find a little concerning…  **Patient: Extensive Progressive**  But [partner] may need support as a carer… Practical, she’s good. She’s good. Very good. Emotional, she seems very subdued… I don’t think she’s hit a problem like this. And the children have been quiet as well…so emotionally I think [partner] may be a little bit keeping it close to her chest. |
| --- |
