# Supplementary material for: The care needs of patients with idiopathic pulmonary fibrosis and their carers (CaNoPy): results of a qualitative study
Source: BMC Pulm Med. 2015 Dec 4;15:155. doi: 10.1186/s12890-015-0145-5 (PMC4670492; doi:10.1186/s12890-015-0145-5)
Supplement: Additional file 10: — Box 9. Carers. (DOCX 13 kb) [file 12890_2015_145_MOESM10_ESM.docx]

PULM-D-15-00026R1

The Care Needs of patients with Idiopathic Pulmonary Fibrosis and their Carers (CaNoPy): results of a qualitative study.

**Box 9. Carers**

**Carer: Limited Stable**

… he [patient] came upstairs and he’d washed the kitchen floor for me and he was… he was finished. He was exhausted. And I felt terrible. I felt awful ‘cos he’d washed the kitchen floor but he felt good ‘cos he’d done it but then he came upstairs and he was white and his eyes were sunken in and… Which is a lot to cope with as well isn’t it, if you’re feeling guilty about things as well. And I did feel guilty. But I thought oh good, I don’t have to do it.

**Carer: Limited Progressive**

It’s the feeling of you know, what is there, you know I wish there was something you could tell me that I could do to assist rather than just sitting there you know. And actually asking “Can I help” makes it worse I think because he just becomes even more anxious then. So I tend to sit there and say nothing and I’m thinking this is awful, I’m sitting here doing nothing and saying nothing because I don’t know quite what to do or say that would be of any assistance you know.

**Male carer and female patient: Limited Stable**

Carer: Well I never used to do anything in the house. Domesticity was girls work… Nothing to do with me, that’s women’s work. That’s how I was brought up and you know. And [patient] was brought up in the same environment, that men don’t do that. That’s nothing to do with them. Kitchens, housework, nothing to do with them it’s all… That’s how she was brought up. But, it’s changed…

Patient: You do everything now and I do nothing.

Carer: It’s changed a lot. A lot.
